# Progress in Surgery

**Published:** 1902-03-22

**Authors:** 


### Progress in Surgery.

### SURGERY OF THE KIDNEY.

**Movable Kidney.**—Henry Morris [1] has considered this affection fully. Some of his more striking observations we abstract. A kidney must be regarded as being abnormally movable under the following circumstances: (1) When the whole kidney descends during deep inspiration below the fingers on bimanual palpation. (2) When it so descends that the greater part can be felt. (3) When the lower half so descends and can be felt. In many of the second class, and all of the first, the kidney can be retained between the fingers even with deep expiration. (4) When the kidney is often out of position during natural respiration; in some of these it may project under the abdominal wall. (5) When the kidney moves in a "cinder-sifting" manner on the posterior abdominal wall without dropping forwards and inwards. The frequency of occurrence of movable kidney has been variously estimated between 1 and 46 per cent. in the female sex. These variations in the estimate are due to cases being overlooked in the post-mortem room, and also in the living subject, and to the fact that physicians at health resorts, who see numbers of hysterical and dyspeptic patients, and examine them carefully for movable kidney, naturally meet with a greater number of patients with this affection than other practitioners. The methods of examination are next described by Morris. Sometimes it is advantageous to place the patient in the knee-elbow or upright position; but, as a rule, it is better to get the patient to roll over on to the sound side and examine bimanually if ordinary examination fails. The kidney is felt at the end of inspiration and the beginning of expiration in this method. Glénard uses the fingers and thumb of one hand mainly in the examination, and claims to feel the kidney at the beginning and throughout the act of inspiration. Israel has the patient lying on the sound side and facing him and uses both hands. The kidney, though very movable, may be quite beyond the reach of palpation. Morris makes it a rule never to declare that a kidney is not movable as a result of a *single examination.* More than one examination should always be made if the condition is suspected. An ectopic kidney may not move with respiration as it may lie too far away from the diaphragm. During the catamenial period the kidney may get very large and new growth be simulated. The chief symptoms are: (1) pain; (2) digestive troubles; (3) neurasthenia, these may occur separately or combined; comparatively rarely (4) changes in the urine are found; and more rarely still (5) symptoms from dragging or compression, such as intestinal obstruction, jaundice, or gastric dilatation. Sometimes the pain increases for some hours on lying down, in others it is much easier then. Renal paroxysms may occur with intense abdominal pain and collapse, and these symptoms are probably due to torsion. Acute hydronephrosis may be produced. The gastric symptoms may be exacerbated leading to "crises" with violent nausea, vomiting, colic, and abdominal distension and tenderness. Jaundice may arise from dragging on the duodenum. Similarly, gastric dilatation may be caused. Kuttner speaks of frequent straining to urinate and polyuria as possible symptoms, and Morris has seen several cases in which there was slight pyuria or hæmaturia and frequent micturition. In diagnosis probably the commonest error is to mistake an enlarged gall-bladder for a "floating" kidney. The two conditions often co-exist. As to treatment, Morris considers belts and pads to be practically useless in uncomplicated cases. It is when enteroptosis is associated with the movable kidney that belts are sometimes useful, and they should not have special renal pads. Next to no value is attached to rest in the recumbent position as a method of treatment. Morris employs one of three operations, viz., Vulliet's, one he calls his own, and a modification of Tuffier's. If both kidneys require fixing, the second should be operated upon a week after the first. G. M. Edebohls (New York) [2] states that the frequent co-existence of movable right kidney and chronic appendicitis often call for nephropexy and appendicectomy on the same patient. The operations should be performed at one sitting, through one and the same lumbar incision. He has done the operation 53 times. It has been his frequent practice, also, after having opened the peritoneum to remove the appendix, to explore the gall-bladder and bile ducts by palpation or even inspection, and this can be done from the same lumbar incision. The writer also states that he has abandoned the use of sutures passing through the kidney substance owing to the glomerular and tubular destruction they caused, and to the fact that such sutures occasionally, by fracturing the kidney, led to the formation of urinary fistulæ. He now removes all the fatty capsule over the convexity of the kidney; reflects the capsule proper on either side to about the middle of the anterior and posterior surfaces of the kidney; sutures the kidney thus laid bare to the quadratus lumborum with four sutures passed through both the reflected and the still attached capsule without penetrating the kidney substance, and closes the wound without drainage. He had operated on 193 patients, and in 68 of them had fixed both kidneys. Three patients had died, giving a mortality of 1·55 per cent. Appendicectomy had been done in 53 of the cases. The results as far as regards permanent anchorage of the kidney had been most gratifying. Roskam [3] states that if a movable kidney first develops in the course of neurasthenia or hysteria it is most important to treat the latter, and only operate on the kidney if the other treatment fails. On the other hand, if the mobility of the kidney existed before the hysteria developed, operation should be performed as soon as possible, as otherwise it will favour the continuance of the neurosis. P. Trekaki,[4] in examining 100 unselected Arab women, including nine virgins, for movable kidney, found the condition present in 41 of them. In thirty the lower third of the kidney was palpable, in nine the lower two-thirds, and in two the whole kidney came down. Thirty-eight of the women had born children and two had not. The fact that the Arabian women do not wear corsets or tight or heavy clothing excludes these as causative factors. Repeated pregnancies, malnutrition and other causes are more in evidence. Swinford Edwards[5] relates the case of a woman aged 22 upon whom he performed nephrectomy for a hydronephrosis which had developed after nephropexy performed a month previously. The pelvis of the kidney was found to be enormously dilated. After drawing out the kidney onto the loin, a probe was easily passed from it down the ureter into the bladder. The hydronephrosis was concluded to have been caused by a kink in the ureter. Possibly a cause for this rare result of nephropexy was that the kidney was fixed too low down, and not, as it ought to be, with its upper half on a level with the lower two ribs.

1 Lancet, Nov. 30, 1901, p. 1469. 2 Med. Rec., Oct. 19, 1901, p. 634. 3 Vide Centralb. f. Chir., Nov. 2, 1901, p. 1085. 4 Vide Centralb. f. Chir., Oct. 5, 1901, p. 989. 5 Med. Press, Sept. 25, 1901, p. 335.

